# Meeting abstracts from the Annual Conference on Hereditary Cancers 2016: Szczecin, Poland. 14-15 September 2016

**DOI:** 10.1186/s13053-017-0081-x

**Published:** 2017-12-05

**Authors:** C. Cybulski, W. Kluźniak, T. Huzarski, D. Wokołorczyk, A. Kashyap, A. Jakubowska, M. Szwiec, T. Byrski, T. Dębniak, B. Górski, V. Sopik, M. R. Akbari, P. Sun, J. Gronwald, S. A. Narod, J. Lubiński, T. Dębniak, D. Dymerska, G. Kurzawski, J. Lubiński, D. Dymerska, K. Tutlewska, M. Kuswik, H. Rudnicka, R. J. Scott, R. Billings, A. Pławski, J. Lubinski, G. Kurzawski, T. Gromowski, K. Kąklewski, W. Marciniak, K. Durda, M. Lener, G. Sukiennicki, K. Kaczmarek, K. Jaworska-Bieniek, K. Paszkowska-Szczur, P. Waloszczyk, J. Lubiński, T. Dębniak, J. Gronwald, K. Hemminki, A. Försti, T. Huzarski, J. Gronwald, C. Cybulski, O. Oszurek, M. Szwiec, K. Gugała, M. Stawicka, Z. Morawiec, T. Mierzwa, M. Falco, H. Janiszewska, E. Kilar, E. Marczyk, B. Kozak-Klonowska, M. Siołek, D. Surdyka, R. Wiśniowski, M. Posmyk, P. Domagała, T. Byrski, P. Sun, J. Lubiński, S. A. Narod, Polish Breast Cancer Consortium, E. N. Imyanitov, K. Kaczmarek, M. Muszyńska, W. Marciniak, G. Sukiennicki, M. Lener, K. Durda, K. Jaworska-Bieniek, T. Gromowski, K. Prajzendanc, N. Peruga, T. Huzarski, T. Byrski, J. Gronwald, C. Cybulski, T. Dębniak, A. Morawski, A. Jakubowska, J. Lubiński, M. R. Lener, R. J. Scott, W. Kluźniak, J. Gronwald, P. Baszuk, C. Cybulski, A. Wiechowska-Kozłowska, T. Huzarski, J. Kładny, S. Pietrzak, A. Soluch, A. Jakubowska, J. Lubiński, A. Plawski, K. Prajzendanc, A. Jakubowska, J. Lubiński, U. R. Rashid, H. Naeemi, N. Muhammad, J. Lubiński, A. Jakubowska, A. Loya, M. A. Yusuf, A. Savanevich, O. Aszurek, J. Gronwald, J. Lubiński, A. Mathe, M. Wong-Brown, W. Locke, C. Stirzaker, S. G. Braye, J. F. Forbes, S. Clark, K. Avery-Kiejda, R. J. Scott, J. Tomiczek-Szwiec, T. Huzarski, M. Szwiec, J. Gronwald, C. Cybulski, E. Marczyk, J. Jakubowicz, E. Kilar, R. Sibilski, M. Stawicka, Z. Morawiec, T. Mierzwa, M. Falco, H. Janiszewska, B. Kozak-Klonowska, M. Siołek, D. Surdyka, R. Wiśniowski, R. Posmyk, P. Domagała, J. Lubiński, M. Szwiec, J. Tomiczek-Szwiec, T. Huzarski, C. Cybulski, J. Lubiński

**Affiliations:** 1 International Hereditary Cancer Center, Department of Genetics and Pathology, Pomeranian Medical University, Szczecin, Poland; 2 Regional Oncology Center, Opole, Poland; 3 Women’s College Research Institute, Toronto, ON Canada; 4 Department of Genetics and Pathology, Pomeranian Medical University, Szczecin, Poland; 5 Department of Genetics and Pathology, Pomeranian Medical University, Szczecin, Poland; 6 The University of Newcastle and Hunter Medical Research Institute, Newcastle, Australia; 7 Division of Genetics, Hunter Area Pathology Service, Newcastle, Australia; 8 Institute of Human Genetics, Polish Academy of Sciences, Poznan, Poland; 9 Department of Genetics and Pathology, Pomeranian Medical University, Szczecin, Poland; 10 Read – Gene, S.A., Grzepnica, Poland; 11 International Hereditary Cancer Center, Department of Genetics and Pathology, Pomeranian Medical University, Szczecin, Poland; 12 German Cancer Research Center, Heidelberg, Germany; 13 International Hereditary Cancer Center, Department of Genetics and Pathology, Pomeranian Medical University, Szczecin, Poland; 14 Regional Oncology Center, Opole, Poland; 15 District Specialist Hospital, Olsztyn, Poland; 16 Regional Oncology Center, Poznań, Poland; 17 Regional Oncology Center, Łódź, Poland; 18 Regional Oncology Hospital, Bygdoszcz, Poland; 19 Nicolaus Copernicus University, Bydgoszcz, Poland; 20 District Specialist Hospital, Świdnica, Poland; 21 Regional Oncology Center, Kraków, Poland; 22 Regional Oncology Center, Kielce, Poland; 23 St John’s Cancer Center, Lublin, Poland; 24 Regional Oncology Hospital, Bielsko Biała, Poland; 25 Regional Oncology Center, Białystok, Poland; 26 Department of Pathology, Pomeranian Medical University, Szczecin, Poland; 27 Women’s College Research Institute, Toronto, Ontario Canada; 28 Regional Oncology Hospital, Szczecin, Poland; 29 N.N. Petrov Institute of Oncology, St.-Petersburg, Russia; 30 Department of Genetics and Pathology, International Hereditary Cancer Center, Pomeranian Medical University, Szczecin, Poland; 31 Read – Gene, S.A., Grzepnica, Poland; 32 Department of Genetics and Pathology, International Hereditary Cancer Center, Pomeranian Medical University, Połabska 4, 70-115 Szczecin, Poland; 33 Discipline of Medical Genetics, School of Biomedical Sciences, Faculty of Health, University of Newcastle and The Hunter Medical Research Institute, Newcastle, New South Wales 2308 Australia; 34 Laboratory of Endoscopy, Division of Heath Care Ministry of Internal Affairs and Administration, Jagiellońska 44, 70-382 Szczecin, Poland; 35 Department of General and Oncological Surgery, Pomeranian Medical University, Al. Powstańców Wlkp. 72, 70-111 Szczecin, Poland; 36 Institute of Human Genetics Polish Academy of Sciences, Poznan, Poland; 37 Department of Genetics and Pathology, International Hereditary Cancer Center, Pomeranian Medical University, Szczecin, Poland; 38 Basic Sciences Research, Shaukat Khanum Memorial Cancer Hospital and Research Centre (SKMCH & RC), Lahore, Pakistan; 39 Department of Genetics and Pathology, Pomeranian Medical University, Szczecin, Poland; 40 Department of Pathology, SKMCH & RC, Lahore, Pakistan; 41 Department of Internal Medicine SKMCH & RC, Lahore, Pakistan; 42 Department of Obstetrics and Gynecology, Grodno State Medical University, Grodno, Belarus; 43 Department of Genetics and Pathology, Pomeranian Medical University, Szczecin, Poland; 44 Centre for Information Based Medicine, Hunter Medical Research Institute, Newcastle, Australia; 45 Priority Research Centre for Cancer, School of Biomedical Sciences and Pharmacy, Faculty of Health, University of Newcastle, Newcastle, Australia; 46 Epigenetics Laboratory, Cancer Program, Garvan Institute of Medical Research, Darlinghurst, Australia; 47 Hunter Area Pathology Service, John Hunter Hospital, New Lambton Heights, New South Wales, Australia; 48 Department of Surgical Oncology, Calvary Mater Newcastle Hospital, Australian New Zealand Breast Cancer Trials Group, New South Wales, Australia; 49 Discipline of Medical Genetics, School of Biomedical Sciences, Faculty of Health, University of Newcastle, Newcastle, Australia; 50 Regional Oncology Center, Opole, Poland; 51 International Hereditary Cancer Center, Department of Genetics and Pathology, Pomeranian Medical University, Szczecin, Poland; 52 Regional Oncology Center, Kraków, Poland; 53 Department of Radiotherapy, Center of Oncology – Maria Skłodowska-Curie Memorial Institute, Cracow Division, Warsaw, Poland; 54 District Specialist Hospital, Świdnica, Poland; 55 Oncology Diagnostic Center, Zielona Gora, Poland; 56 Regional Oncology Center, Poznań, Poland; 57 Regional Oncology Center, Łódź, Poland; 58 Regional Oncology Hospital, Bygdoszcz, Poland; 59 Regional Oncology Hospital, Szczecin, Poland; 60 Nicolaus Copernicus University, Bydgoszcz, Poland; 61 Regional Oncology Center, Kielce, Poland; 62 St John’s Cancer Center, Lublin, Poland; 63 Regional Oncology Hospital, Bielsko Biała, Poland; 64 Regional Oncology Center, Białystok, Poland; 65 Department of Pathology, Pomeranian Medical University, Szczecin, Poland; 66 Regional Oncology Center, Opole, Poland; 67 Depertment of Genetics and Pathology, International Hereditary Cancer Center, Pomerian Medical University, Szczecin, Poland

## A1 New genes associated with a high risk of breast cancer

### Cybulski C^1^, Kluźniak W^1^, Huzarski T^1^, Wokołorczyk D^1^, Kashyap A^1^, Jakubowska A^1^, Szwiec M^2^, Byrski T^1^, Dębniak T^1^, Górski B^1^, Sopik V^1^, Akbari MR^3^, Sun P^3^, Gronwald J^1^, Narod SA^3^, Lubiński J^1^

#### ^1^International Hereditary Cancer Center, Department of Genetics and Pathology, Pomeranian Medical University, Szczecin, Poland; ^2^Regional Oncology Center, Opole, Poland; ^3^ Women’s College Research Institute, Toronto, ON, Canada

We tested over 20, 000 genes by whole-exome sequencing in 144 Polish women with breast cancer from families with strong aggregation of this tumor. We identified a new breast cancer susceptibility gene (RECQL). In Poland, there is one major founder mutations of RECQL. Our results suggest that the risk of breast cancer among the carriers is increased over 5-fold. In addition, we detected two PALB2 founder mutations in our population which predispose to poor prognosis breast cancer. Over half of breast cancer patients with a PALB2 mutation died within 10 years from the diagnosis. The survival among women with breast cancer caused by mutations of PALB2 strongly depended on tumor size. Among mutation carriers with tumors smaller than 2 cm, 10-year survival was 82%. In contrast, among mutation carriers with tumors greater than 2 cm 10-year survival was only 32%.

In summary, our research enabled to identify new determinants (inherited mutations) for breast cancer, the most common malignant tumor in women.

**Acknowledgements**

The study was funded by the Polish National Science Centre (DEC- 2011/03/N/NZ2/01510).

## A2 Effectiveness of detection of mutations within genes predisposing to Lynch Syndrome

### Dębniak T, Dymerska D, Kurzawski G, Lubiński J

#### Department of Genetics and Pathology, Pomeranian Medical University, Szczecin, Poland

In diagnosis of predisposition to hereditary non-polyposis colorectal cancer (Lynch syndrome) resulting from inheritance of mutations within mismatch repair genes (MMR) it is appropriate to apply techniques in order allowing the most effective detection of these mutations. According to our experience (International Hereditary Cancer Center, PMU) which is consistent with literature data, the basic criteria for the implementation of DNA testing are pedigree and clinical data. In Poland testing based on analysis of recurrent mutations, characteristic for our population, should be performed as the first. Then, among non-carriers of these mutations it seems to be appropriate to analyse occurrence of large rearrangements with MLPA and MMR genes expression with IHC testing. Next generation sequencing of MMR genes should be performed in cases with IHC abnormalities. The use of different protocols based upon IHC testing and sequencing in sporadic CRC results in huge increase of costs- up to a level of 150 thousand PLN for one mutation detection.

## A3 New EPCAM founder mutation in ZXSDGPolish population

### Dymerska D^1^, Tutlewska K^1^, Kuswik M^1^, Rudnicka H^1^, Scott RJ^2^, Billings R^3^, Pławski A^4^, Lubinski J^1^, Kurzawski G^1^

#### ^1^Department of Genetics and Pathology, Pomeranian Medical University, Szczecin, Poland; ^2^The University of Newcastle and Hunter Medical Research Institute, Newcastle, Australia; ^3^Division of Genetics, Hunter Area Pathology Service, Newcastle, Australia; ^4^Institute of Human Genetics, Polish Academy of Sciences, Poznan, Poland

The main recognized founder populations in the world are those of Finland, Sardinia, Iceland, Costa Rica, the northern Netherlands and several discrete ethic groups, including the Ashkenazi Jews. A country where there are unequivocal founder mutations is Poland where a substantial fraction of hereditary breast and prostate cancers may be explained by founder mutations in BRCA1, RECQL, PALB2, NBS1 and CHEK2 genes, respectively. For hereditary colorectal cancer (CRC), implication of Polish founder mutation has been proved only once for one of MMR (mismatch repair) genes - MLH1 (which causes Lynch syndrome (LS) when mutated). Lately, we have revealed new founder mutation in another LS-related gene – EPCAM (TACSTD1). The mechanism of pathogenesis of EPCAM caused-LS is different comparing to single germline mutation in MMR genes. Mutation results in loss of last 3’ exons, leading to transcription read-through and inactivation of downstream gene – MSH2.

Presence of founder mutation provides an opportunity to design an inexpensive test which can be applied as a screening method. We designed simple PCR reaction, examined large group of CRCs patients and characterized groups where EPCAM founder diagnostics is the most effective.

## A4 Vitamin D concentration and frequent variants of VDR gene as a markers of detection probability of breast, lung, prostate and colorectal cancers

### Gromowski T^1^, Kąklewski K^2^, Marciniak W^1^, Durda K^1^, Lener M^1^, Sukiennicki G^1^, Kaczmarek K^1^, Jaworska-Bieniek K^1^, Paszkowska-Szczur K^1^, Waloszczyk P^1^, Lubiński J^1^, Dębniak T^1^

#### ^1^Department of Genetics and Pathology, Pomeranian Medical University, Szczecin, Poland; ^2^Read – Gene, S.A., Grzepnica, Poland

**Aim of study:**

The aim of this study was to evaluate the association between serum vitamin D3 concentration, common variants of VDR gene and the number of breast, lung, prostate and colorectal cancers.

Material and methods:

The study was conducted in 4 groups:

Group I - 200 unselected patients with breast cancer and 400 controls.

Group II - 200 unselected patients with lung cancer and 400 paired controls.

Group III - 200 unselected patients with prostate cancer and 400 controls.

Group IV - 195 unselected patients with colorectal cancer and 390 controls.

Groups 25(OH)D3 levels were determined by high performance liquid chromatography - HPLC. 5 common VDR variants: rs2228570/FokI, rs1544410/BsmI, rs7975232/ApaI, rs731236/TaqI, rs11568820/CDX2 were genotype using TaqMan assays.

Results:

In the study we observed that low 25(OH)D3 concentration (0-12 ng/ml) was associated with increased lung cancer incidence (OR 2,13; p=0,01), regardless of VDR genotype. In group of prostate cancer patients we found statistically significant decrease risk of cancer for individuals with low 25(OH)D3 concentration (0-20 ng/ml) and genotype CC of variant rs1544410/BsmI (OR 0,42; p=0,02) or AA genotype of variant rs731236/TaqI (OR 0,40; p=0,006).

## A5 A population screening - detection of BRCA1 and other genes founder mutations

### Gronwald J

#### International Hereditary Cancer Center, Department of Genetics and Pathology, Pomeranian Medical University, Szczecin, Poland

Effective screening program must relate to important health problem, reach out to the entire population and be rational from an economic point of view. In most countries genetic tests, which allow diagnosis of high hereditary predisposition to cancer are applied in a strictly selected group of patients. This practice is caused by high costs of genetic testing. Methods of patients eligibility for a genetic testing are based mostly on meeting the relevant pedigree criteria or clinical symptoms. Due to incomplete gene penetration, the increasingly small number of family members, or lack of knowledge of cancer family history of ancestors such strategy make impossible to diagnose about half of mutation carriers who do not meet the inclusion criteria. In populations with domination of recurrent founder mutations strategies focusing on testing of a limited number of selected mutations allow significantly reduce the cost of genetic testing and applying them to the wide population. In 1999, three founder mutations in the BRCA1 gene, which account for about 90% of all detectable mutations were determined in the Polish population. In this study we present the experience to date with the diagnosis of high hereditary predisposition to cancer in the context of genetic testing and discuss the conditions necessary for carrying out the population wide genetic screening.

## A6 Epidemiology and genetics of familial cancer

### Hemminki K, Försti A

#### German Cancer Research Center, Heidelberg, Germany

Based on a family database, such as the Swedish Family-Cancer Database, one can estimate that somewhat more than 10% of all cancers are familial, i.e., at least 2 first-degree relatives are diagnosed with the same cancer. However, because of low penetrance, sex-specific cancers and small families, this figure is scientifically an underestimate of the scope of familial risk, and psychologically, familial cancer has a far larger dimension. Practically all families have cancer patients and, as 25% of all deaths are due to cancer, most families have suffered a cancer death. Cancer diagnosis in a family member is a dramatic event and it is even more so if the patient dies in a short time. It is true that most relatives of cancer patients have no familial risk, as most cancers are sporadic. This is however not known at the time of the family member’s diagnosis and the empirical data predicts an approximately 2-fold risk to a healthy first-degree relative from a single case family. The risk is substantially higher, say 5 to 15-fold depending on cancer type, for a healthy individual if two or more of their close relatives are diagnosed with the same cancer. So far the focus has been on same cancer in family members but our new data show that even different cancers in family members may signal a familial risk.

Known susceptibility genes are estimated to explain some 30% of the familial clustering of breast, prostate and colorectal cancers but much less of the familial clustering of lung cancer. The recently identified low-penetrance genes/loci explain a large proportion of cancer occurrence (population-attributable fraction) but they explain only a small proportion of the known familial risks for these cancers. We have collaborated with Jan Lubinski’s team in developing a pipeline for analyzing germline genomes from Mendelian types of pedigrees (Försti et al. Pedigree based DNA sequencing pipeline for germline genomes of cancer families.

Hered Cancer Clin Pract. 2016 Aug 9;14:16. The variant calling step distinguishes three types of genomic variants: single nucleotide variants (SNVs), indels and copy number variants (CNVs), which undergo technical quality control. Mendelian types of variants are assumed to be rare and usually variants with frequencies higher that 0.1% are screened out. Segregation in the pedigree allows variants to be present in affected family members and not in old unaffected ones. The effectiveness of variant segregation depends on the number and relatedness of the family members; if over 5 third-degree (or more distant) relatives are available the experience has shown that the number of likely variants is reduced from many hundreds to a few tens. These are then subjected to bioinformatic analysis, starting with the combined annotation dependent depletion (CADD) tool, which predicts the likelihood of the variant being deleterious. Different sets of tools are used for coding variants, 5’- and 3’-UTRs, and intergenic variants. The likelihood of success of the present genomic pipeline in finding novel high- or medium-penetrant genes depends on many steps but first and foremost, the pedigree needs to reasonably large and the assignments and diagnoses among the members need to be correct.

## A7 The impact of oophorectomy on survival after breast cancer in BRCA1-positive breast cancer patients

### Huzarski T^1^, Gronwald J^1^, Cybulski C^1^, Oszurek O^1^, Szwiec M^2^, Gugała K^3^, Stawicka M^4^, Morawiec Z^5^, Mierzwa T^6^, Falco M^16^, Janiszewska H^7^, Kilar E^8^, Marczyk E^9^, Kozak-Klonowska B^10^, Siołek M^10^, Surdyka D^11^, Wiśniowski R^12^, Posmyk M^13^, Domagała P^14^, Byrski T^1^, Sun P^15^, Lubiński J^1^, Narod SA^15^; Polish Breast Cancer Consortium

#### ^1^International Hereditary Cancer Center, Department of Genetics and Pathology, Pomeranian Medical University, Szczecin, Poland; ^2^Regional Oncology Center, Opole, Poland; ^3^District Specialist Hospital, Olsztyn, Poland; ^4^Regional Oncology Center, Poznań, Poland; ^5^Regional Oncology Center, Łódź, Poland; ^6^Regional Oncology Hospital, Bygdoszcz, Poland; ^7^Nicolaus Copernicus University, Bydgoszcz, Poland; ^8^District Specialist Hospital, Świdnica, Poland; ^9^Regional Oncology Center, Kraków, Poland; ^10^Regional Oncology Center, Kielce, Poland; ^11^St John's Cancer Center, Lublin, Poland; ^12^Regional Oncology Hospital, Bielsko Biała, Poland; ^13^Regional Oncology Center, Białystok, Poland; ^14^Department of Pathology, Pomeranian Medical University, Szczecin, Poland; ^15^Women’s College Research Institute, Toronto, Ontario, Canada; ^16^Regional Oncology Hospital, Szczecin, Poland

The aim of the study is to identify treatments which predict survival for women with a BRCA1 mutation, including oophorectomy and chemotherapy. 476 women with stage I to stage III breast cancer who carried a BRCA1 mutation were followed from diagnosis until April 2015. Information on treatment was obtained from chart review and patient questionnaires. Dates of death were obtained from the Poland vital statistics registry. Survival curves were compared for different subgroups according to treatment received. Predictors of overall survival were determined using the Cox proportional hazards model. The ten-year overall survival was 78.3 % (95 % CI 74.2-82.6 %) and the ten-year breast cancer-specific survival was 84.2 % (95 % CI 80.5-88.0 %). Sixty-two patients died of breast cancer, 14 patients died of ovarian cancer, and 2 patients died of peritoneal cancer. Oophorectomy was associated with a significant reduction in all-cause mortality in the entire cohort (adjusted HR = 0.41; 95 % CI 0.24-0.69; p = 0.0008) and in breast cancer-specific mortality among ER-negative breast cancer patients (HR = 0.44; 95 % CI 0.22-0.89; p = 0.02). Among women with breast cancer and a BRCA1 mutation, survival is greatly improved by oophorectomy due to the prevention of deaths from both breast and ovarian cancer.

## A8 Old and new drugs for hereditary cancers

### Imyanitov EN

#### N.N. Petrov Institute of Oncology, St.-Petersburg, Russia

There is an impressive progress in the development of specific treatments for hereditary cancers. The most common type of familial cancer syndrome, BRCA1/2-related breast/ovarian cancer (BC/OC), is characterized by a pronounced deficiency in the DNA double-strand break repair by homologous recombination. This property causes high sensitivity of hereditary BC/OC to some common drugs, such as cisplatin, mitomycin C, anthracyclines, etc. PARP-inhibitors represent the first example of targeted therapy, which was specifically developed to treat tumors arising in germ-line mutation carriers. Some case reports and case series suggest potential utility of high-dose chemotherapy for the management of BRCA1/2-driven malignancies. Exceptionally high plasticity of BRCA1/2-related cancers significantly compromises therapeutic interventions resulting in a rapid emergence of resistant clones. MSI-H colorectal tumors, including those arising in the context of HNPCC syndrome, are highly antigenic and therefore can be managed by immune checkpoint inhibitors. There are some other examples of targeted drugs, which are used for the treatment of patients with orphan cancer syndromes. Turnaround time for genetic testing is a critical factor affecting treatment decisions for the patients with hereditary cancers.

## A9 Zinc and copper as risk markers for cancers in polish population

### Kaczmarek K^1*^, Muszyńska M^2*^, Marciniak W^2^, Sukiennicki G^1^, Lener M^1^, Durda K^1^, Jaworska-Bieniek K^1^, Gromowski T^1^, Prajzendanc K^1^, Peruga N^1^, Huzarski T^1^, Byrski T^1^, Gronwald J^1^, Cybulski C^1^, Dębniak T^1^, Morawski A^2^, Jakubowska A^1^, Lubiński J^1,2^

#### ^1^Department of Genetics and Pathology, International Hereditary Cancer Center, Pomeranian Medical University, Szczecin, Poland; ^2^Read – Gene, S.A., Grzepnica, Poland

^*^Authors contributed equally to this work.

**Background**

There is an evidence that trace elements such as zinc and copper influence the process of carcinogenesis. Concentrations of copper and zinc in serum are strictly regulated by the compensation mechanism, which provides a stable level of both elements. The copper to zinc ratio (Cu/Zn) is a potentially diagnostic parameter, more sensitive than analyzing each element separately.

**Aim of the study**

The aim of the study was to evaluate the relationship between zinc and copper blood levels and subsequent cancer risk in a large cohort of persons followed for incident cases of cancer in Szczecin Poland, using a case-control study design.

**Material and methods**

Study group was selected from among persons whose biological material is biobanked in our center. In biobank we collected samples from ~26 000 people with no previous cancer diagnosis at the time of blood collection. All patients were followed for cancers diagnosis. Study group was divided into 2 subgroups: 88 men with all sites cancers and 166 unaffected controls, 123 women with all sites cancers (BRCA1 mutation carriers exclude) and 238 unaffected controls. Zinc level in serum was measured by inductively coupled plasma mass spectrometry (ICP-MS) using Elan DRC-e ICP-Mass Spectrometer, Perkin Elmer. For statistical analysis individuals in each group were divided into 4 quartiles. We performed comparison of number of cases and controls in each quartile. OR was calculated using Fisher’s exact test.

**Results**

For zinc serum level it was observed that women zinc concentration in the range of 780-850 ug/l had an almost 2-fold lower risk of disease compared to women with lower levels of Zn (OR = 0.53; p = 0.02; 95% CI = 0,31-0,91). In group of males no association was observed between serum zinc levels and cancer incidence. For copper serum zinc level there was no association with cancer risk for females and males. Cu/Zn ratio was related to cancer risk. Women diagnosed with cancer had a 3-fold lower risk of disease with Cu/Zn ratio between 1.16 and 1.29 (OR=0,32; p=0,0005; 95% CI=0,16-0,62). For males, Cu/Zn ratio between 1.17-1.31 was associated with an almost 3-fold decrease in cancer risk (OR = 0.37, p = 0.04; 95% CI = 0,16-0,88).

**Conclusions**

Serum zinc concentration is a marker of cancer risk for females. The lowest risk is associated with zinc concentration in the range of 780-850 ug/l. Copper serum concentration alone is not a risk marker. Cu/Zn ratio is a good marker of cancer risk, both for men and women. The lowest risk is associated with Cu/Zn ratio between 1.16 and 1.29 for females and between 1.17-1.31 for males.

**Acknowledgements**

This project was financially supported by National Science Centre, grant no. 2012/07/N/NZ4/02433.

## A10 Do founder mutations characteristic of some cancer sites also predispose to pancreatic cancer?

### Lener MR^1^, Scott RJ^2^, Kluźniak W^1^, Gronwald J^1^, Baszuk P^1^, Cybulski C^1^, Wiechowska-Kozłowska A^3^, Huzarski T^1^, Kładny J^4^, Pietrzak S^1^, Soluch A^1^, Jakubowska A^1^, Lubiński J^1^

#### ^1^Department of Genetics and Pathology, International Hereditary Cancer Center, Pomeranian Medical University, Połabska 4, 70-115 Szczecin, Poland; ^2^Discipline of Medical Genetics, School of Biomedical Sciences, Faculty of Health, University of Newcastle and The Hunter Medical Research Institute, Newcastle, New South Wales 2308, Australia; ^3^Laboratory of Endoscopy, Division of Heath Care Ministry of Internal Affairs and Administration*,* Jagiellońska 44, 70-382 Szczecin, Poland; ^4^Department of General and Oncological Surgery, Pomeranian Medical University, Al. Powstańców Wlkp. 72, 70-111 Szczecin, Poland

**Introduction**

Understanding of the etiology and risk of pancreatic cancer (PaCa) and Familial Pancreatic Cancer (FPC) is still poorly understood. This study evaluated the prevalence of 10 Polish founder mutations in 4 genes among PaCa, FPC patients, and assesses their possible association with the risk of disease in Poland.

**Aim**

The aim of the study was to evaluated the prevalence of 10 Polish founder mutations in 4 genes among:

***1.*** Pancreatic cancer patients - PaCa

***2.*** Individuals from families with FPC syndrome and assesses their possible association with the risk of disease in Poland.

**Material and methods**

In the study, 383 PaCa patients, 398 FPC individuals and 4000 control subjects were genotyped for founder mutations in *BRCA1* (5382insC, 4153delA, C61G), *CHEK2* (1100delC, IVS2 + 1G>A, del5395, I157T), *NBS1* (657del5) and *PALB2* (509_510delGA, 172_175delTTGT) genes.

**Results**

***1.*** A statistically significant association between the 657del5 mutation and an increased risk of pancreatic cancer was observed for *NBS1*. The Slavic *NBS1* mutation (657delACAAA) was detected in 8 of 383 (2,09%) unselected cases compared with 22 of 4000 (0.55%) controls (OR – 3,80, p = 0,002).

The *PALB2* 509_510delGA and 172_175delTTGT mutations combined were seen in 2 (0,52%) unselected cases of PaCa and in 8 (0,20%) of 4000 controls (OR – 2,61, p = 0,49). For *BRCA1,* the three mutations combined were detected in 4 of 383 (1,04%) PaCa patients and in 17 of 4000 (0,42%) controls (OR – 2,46, p = 0,20). *CHEK2* mutations were not associated with the risk of pancreatic cancer (OR – 1,11 p= 0,72).

***2.*** A statistically significant association was observed between the 172_175delTTGT mutation of *PALB2* gene and an increased risk of FPC syndrome (OR-10.05, p=0.048). In addition, we observed an increased risk of cancer in FPC family members with cancer and *BRCA1* mutation (OR-6.72, p=0.006). A novel associations was found between FPC family members with cancer and *CHEK2* mutations (OR-2.26, p=0.008) with a noticeable contribution of missense variant I157T of *CHEK2* (OR-2.17, p=0.026).

**Conclusion**

**1.** The founder mutation in *NBS1* (657del5) was associated with an increased risk of PaCa in heterozygous carriers, indicating that this mutation appears to predispose to cancer of the pancreas. By identifying pancreatic cancer risk groups, founder mutation testing in Poland should be considered for people at risk for PaCa.

**2.** The founder mutations in the genes *BRCA1*, *PALB2* and *CHEK2* cause a small percentage of the familial pancreatic cancer syndrome in the Polish population. Following the confirmation in larger studies, these mutations can be added to the panel of genes tested in families with a diagnosis of FPC syndrome.

## A11 Spectrum of APC gene mutations in Poland

### Plawski A

#### Institute of Human Genetics Polish Academy of Sciences, Poznan, Poland

Familial adenomatous polyposis (FAP) is a well-known hereditary disorder characterized by the occurrence of numerous polyps in patient’s colon and rectum at unusually early ages. The first symptoms of FAP are diarrhea and blood in the stool. Weight loss and weaknesses occur after the development of advanced tumour. The incidence of the FAP disorder is one per 10000 newborns. There are high levels of heterogeneity with regard to the number and timing of the occurrence of polyps. The classical form of FAP is characterized by the presence of more than 100 polyps, which appear in the second decade of life. The average time of occurrence of polyps is 15 years. The polyps have large potential for the development towards malignant tumour. Malignancy can occur from late childhood onwards. Attenuated adenomatous polyposis coli has more benign course of disease in contrast to classical FAP. The occurrence of FAP is associated with mutations in the *APC* tumor suppressor gene, which was described in 1991. Since then, many studies have been done to analyze the distribution of mutations in individual populations and to determine the function of the gene and a diagnostic approach to FAP. Molecular studies of colonic polyposis have been performed in Poland for over twenty years. Here we present result of our APC gene studies including searching for point mutation and CNV occurrence using HRM, C-HRM and MLPA methods in a group of over 700 FAP probants.

## A12 Methylation of *BRCA1* promoter in women with breast cancer in Polish Population

### Prajzendanc K, Jakubowska A, Lubiński J

#### Department of Genetics and Pathology, International Hereditary Cancer Center, Pomeranian Medical University, Szczecin, Poland

Methylation of CpG islands in DNA is an epigenetic modification that causes silencing of genes and might be associated with cancer risk if present in peripheral blood. It has been shown that constitutional methylation of *BRCA1* promoter correlates with breast cancer risk, especially with triple-negative tumours. In this part of the study we evaluated frequency of methylation of *BRCA1* promoter in peripheral blood among women with triple-negative breast cancer. We examined 508 TNBC cases. All women were negative for *BRCA1* germline mutations. Methylation in all samples was measured using methylation-sensitive high-resolution melting (MS-HRM). Samples with any detectable methylation level were considered as positive. Methylation of *BRCA1* promoter was present in 223 of 508 women (43,9%). Obtained results confirm previous studies, in particular the frequency of methylation in women with TNBC in our pilot study. In future, analysis will be performed among unselected breast cancer cases and healthy controls groups in order to assess the correlation of *BRCA1* promoter methylation with breast cancer risk.

**Acknowledgements**

The study was supported by the National Science Centre (NCN) grant 2014/15/B/NZ1/03386.

## A13 Contribution of MLH1, MSH2 and MSH6 germline mutations to colorectal cancer in Pakistan

### Rashid UR^1^, Naeemi H^1^, Muhammad N^1^, Lubiński J^2^, Jakubowska A^2^, Loya A^3^, Yusuf MA^4^

#### ^1^Basic Sciences Research, Shaukat Khanum Memorial Cancer Hospital and Research Centre (SKMCH & RC), Lahore, Pakistan; ^2^Department of Genetics and Pathology, Pomeranian Medical University, Szczecin, Poland;^3^Department of Pathology, SKMCH & RC, Lahore, Pakistan; ^4^Department of Internal Medicine SKMCH & RC, Lahore, Pakistan

**Background**

Germline mutations in the DNA mismatch repair (MMR) genes account for the majority of hereditary non-polyposis colorectal cancer (HNPCC). Since nothing is known about HNPCC and the contribution of MMR genes mutation to colorectal cancer (CRC) in Pakistan, we investigated the prevalence of MLH1, MSH2, and MSH6 mutations in CRC patients from this population.

**Materials and methods**

Consecutive CRC cases (n=212) were recruited at the SKMCH&RC, between November 2007 to March 2011. Clinico-histopathological data, family history and blood samples were collected. Cases fulfilling the Amsterdam-II criteria or a less stringent criteria were classified as HNPCC/suspected-HNPCC (Group 1; n=29). Others cases were designated as non-HNPCC (Group 2; n=183). MLH1, MSH2 and MSH6 genes were comprehensively screened using denaturing high-performance liquid chromatography followed by DNA sequencing of variant fragments in Group 1. Deleterious mutations identified in Group 1 were subsequently screened in Group 2.

**Results**

Seven distinct pathogenic MLH1/MSH2 mutations were identified in ten patients of Group 1 (10/29; 34.5%), one of these was novel. Two recurrent mutations, c.1358dup MLH1 and c.943-1G>C MSH2 were identified in two patients of Punjabi ethnicity or three cases of Pathan background, respectively. A novel c.2656G>T MSH2 mutation was detected in a female patient diagnosed with endometrial and breast cancer from a family with phenotypic overlap of hereditary breast and ovarian cancer and suspected-HNPCC. Disease-causative MSH6 mutations were not detected. Screening of the 183 non-HNPCC patients in Group 2 for the seven MLH1/MSH2 mutations revealed one additional patient harboring MLH1 mutation (1/183; 0.5%). The MLH1/MSH2 mutation carriers were more often presented with proximal tumor (5/10, 50% vs. 27/200, 13.5%; p = 0.02) and greater tumor size (>5 cm) (6/10, 60% vs. 28/200, 14.0%; p = 0.02) than non-carriers.

**Conclusion**

Our findings show that MLH1 and MSH2 mutations account for a substantial proportion of HNPCC/suspected-HNPCC patients in Pakistan. The present study warrants the screening of MLH1 and MSH2 genes in HNPCC/suspected-HNPCC families from Pakistan.

## A14 BRCA1-associated ovarian cancer in Belarus: towards a more complete picture

### Savanevich A^1^, Aszurek O^2^, Gronwald J^2^, Lubiński J^2^

#### ^1^Department of Obstetrics and Gynecology, Grodno State Medical University, Grodno, Belarus; ^2^Department of Genetics and Pathology, Pomeranian Medical University, Szczecin, Poland

The problem of hereditary cancer is urgent and needs new approaches to the cure of this group of patients. The aim of the work was the study the frequency of emergence in the Republic of Belarus the three main "founder" mutations in gene BRCA1 (5382insC, C61G, 4154delA) in the patients with ovarian cancer and clinical peculiarities of ovarian tumor of its carriers. For estimating the role of hereditary predisposition in development of ovarian cancer were carried out 169 investigations of DNA samples in the patients with newly diagnosed ovarian cancer after surgical treatment, unselected for age or family history, who were under treatment in Grodno region in 2008- 2011 year. There were determined three main hereditary «founder» mutations BRCA1: 5382insC, 4153delA, C61G. In 25 patients with ovarian cancer were exposed mutations in BRCA1 gene: 15 mutations 5382insC (46%), 8 mutations 4153delA (32%) and 2 mutations C61G (8%). The age of the patients with hereditary ovarian cancer at the moment of stating the diagnosis varied from 38 to 79 years old. Family cancer history was present with 13 patients (46%), however, only 31% of them had relatives with ovarian cancer and/or breast cancer. In 5 out of 25 women (20%) took place primary multiple cancer. Surgical treatment was carried out for 27 (96%) patients. In the majority of cases Т3NхМ0 stage was diagnosed, both ovaries were affected and the tumors were of big size (up to 50 cm in diameter). According to the results of histological investigation all the tumors of mutation holders in BRCA1 gene were high-grade serous adenocarcinomas. The carried out analyses of overall survival and progression-free survival showed that the surveillance of patients with advanced forms of hereditary ovarian cancer is higher than in general population.

Hereditary ovarian cancer in Belarus is associated with the mutation in BRCA1 gene, in 78% it is connected with 5382insC and 4153delA mutations. Hereditary ovarian cancer is characterized by the fast growth of tumors and high-grade differentiation, both sided disease of ovaries, high frequency of primary multiple cancer.

## A15 DNA methylation profile of triple negative breast cancer-specific genes comparing lymph node positive patients to lymph node negative patients

### Mathe A^1,2^, Wong-Brown M^1^, Locke W^3^, Stirzaker C^3^, Braye SG^4^, Forbes JF^1,5^, Clark S^3^, Avery-Kiejda K^1,2^ and Scott RJ^1,6^

#### ^1^Centre for Information Based Medicine, Hunter Medical Research Institute, Newcastle, Australia; ^2^Priority Research Centre for Cancer, School of Biomedical Sciences and Pharmacy, Faculty of Health, University of Newcastle, Newcastle, Australia; ^3^Epigenetics Laboratory, Cancer Program, Garvan Institute of Medical Research, Darlinghurst, Australia; ^4^Hunter Area Pathology Service, John Hunter Hospital, New Lambton Heights, New South Wales, Australia; ^5^Department of Surgical Oncology, Calvary Mater Newcastle Hospital, Australian New Zealand Breast Cancer Trials Group, New South Wales, Australia; ^6^Discipline of Medical Genetics, School of Biomedical Sciences, Faculty of Health, University of Newcastle, Newcastle, Australia

**Introduction**

Triple negative breast cancer (TNBC) is the most aggressive breast cancer subtype with no targeted treatment available. Our previous study identified 38 TNBC-specific genes with altered expression comparing tumour to normal samples. This study aimed to establish whether DNA methylation contributed to these expression changes in the same cohort as well as if disease progression from primary breast tumour to lymph node metastasis was associated with changes in the epigenome.

**Methods**

We obtained DNA from 23 primary TNBC samples, 12 matched lymph node metastases, and 11 matched normal adjacent tissues and assayed for differential methylation profiles using Illumina HumanMethylation450 BeadChip arrays. The results were validated in an independent cohort of 70 primary TNBC samples.

**Results**

The expression of 16/38 TNBC-specific genes was associated with alteration in DNA methylation. Novel methylation changes between primary tumours and lymph node metastases, as well as those associated with survival were identified. Altered methylation of 18 genes associated with lymph node metastasis were identified and validated.

**Conclusion**

This study reveals the important role DNA methylation plays in altered gene expression patterns of TNBC-specific genes and lymph node metastases. The novel insights into progression of TNBC to secondary disease may provide potential prognostic indicators for this hard-to-treat breast cancer subtype.

## A16 Oophorectomy in the treatment of breast cancer in carriers of CHEK2 mutation

### Tomiczek-Szwiec J^1^, Huzarski T^2^, Szwiec M^1^, Gronwald J^2^, Cybulski C^2^, Marczyk E^3^, Jakubowicz J^4^, Kilar E^5^, Sibilski R^6^, Stawicka M^7^, Morawiec Z^8^, Mierzwa T^9^, Falco M^10^, Janiszewska H^11^, Kozak-Klonowska B^12^, Siołek M^12^, Surdyka D^13^, Wiśniowski R^14^, Posmyk R^15^, Domagała P^16^, Lubiński J^2^

#### ^1^Regional Oncology Center, Opole, Poland; ^2^International Hereditary Cancer Center, Department of Genetics and Pathology, Pomeranian Medical University, Szczecin, Poland; ^3^Regional Oncology Center, Kraków, Poland; ^4^Department of Radiotherapy, Center of Oncology – Maria Skłodowska-Curie Memorial Institute, Cracow Division, Warsaw, Poland; ^5^District Specialist Hospital, Świdnica, Poland; ^6^Oncology Diagnostic Center, Zielona Gora, Poland; ^7^Regional Oncology Center, Poznań, Poland; ^8^Regional Oncology Center, Łódź, Poland; ^9^Regional Oncology Hospital, Bygdoszcz, Poland; ^10^Regional Oncology Hospital, Szczecin, Poland; ^11^Nicolaus Copernicus University, Bydgoszcz, Poland; ^12^Regional Oncology Center, Kielce, Poland; ^13^St John's Cancer Center, Lublin, Poland; ^14^Regional Oncology Hospital, Bielsko Biała, Poland; ^15^Regional Oncology Center, Białystok, Poland; ^16^Department of Pathology, Pomeranian Medical University, Szczecin, Poland

**Introduction**

Four founder mutations of CHEK2 gene were found in the Polish population. Three mutations shorten protein del5395, IVS2 + 1G> A, 1100delC, and one is a single nucleotide substitution leading to amino acid substitution (missense mutation I157T). Mutations shortening protein are associated with a higher risk of breast cancer (approximately 3 times higher) than mutation I157T (risk increased about 1.5 times). Survival rating of patients with breast cancer and with one of the four mutations in the CHEK2 gene showed no significant differences compared to the group without the mutation. In the case of patients with a genetic breast cancer associated with mutations in the BRCA1 gene oophorectomy improves survival.Oophorectomy role in women with breast cancer related to mutations in CHEK2 gene has not yet been determined.

**Materials and methods**

The aim of this study was to evaluate the effect of oophorectomy on the survival in women with breast cancer associated with mutations in CHEK2 gene. The study included 11,570 women with invasive breast cancer in stage I to III recognized in the years 1997 to 2012 in 15 centers in Poland. Patients diagnosed up to the of 50 were 63.7% of the whole group (7368/11570). One of the four mutations of CHEK2 gene was detected in 1047 women (9.0%). The analysis of survival was performed in a group of 984 who obtained data about oophorectomy. Patients were prospectively observed from the time of diagnosis to August 2016. Data on clinical and histopathologic features of breast cancers, and data about oophorectomy were obtained on the basis of the available medical records. Information about death was obtained on the basis of data from the Ministry of Internal Affairs and Administration. There was made a comparison of survival in patients with and without oophorectomy. Survival was estimated by the Kaplan-Meier method based on the program STATISTICA12. There was conducted a separate analysis for mutations shortening protein and missense type. Oophorectomy was performed in 191 patients (19.4%). Average time of observation was 8.17 years.

**Results**

In assessed observation time, the survival time in the group of patients with mutations shortening protein and with oophorectomy amounted 86.4% compared to 73.3% in the group without oophorectomy (p = 0.009). In a subgroup of patients up to the age of 50 oophorectomy had an influence on higher rate of survival (88.1% vs 72.7%; p = 0.02), and over the age of 50 it had not (83.3% vs 74.2%; p = 0.3). A similar result was obtained for mutation missense where the whole group had an influence on survival (82.5% vs 74.9%; p = 0.01). The oophorectomy significantly decreased mortality in the subgroup up to the age of 50 with recognition (85.9% vs 75.4%; p = 0.006) and had no effect in elderly patients (77.1% vs 78.3%; p = 0.7). The results show the impact of oophorectomy on prognosis in young women with breast cancer and the presence of mutations in CHEK2 gene. The presented study was multicenter, prospective, observational. This is the first work, which showed the influence of oophorectomy for improvement of survival in carriers of mutations in CHEK2 gene diagnosed with breast cancer.

## A17 The type of recurrence in patients diagnosed with breast cancer and founder mutations in CHEK2 gene – experience of one center

### Szwiec M^1^, Tomiczek-Szwiec J^1^, Huzarski T^2^, Cybulski C^2^, Lubiński J^2^

#### ^1^Regional Oncology Center, Opole, Poland; ^2^Depertment of Genetics and Pathology, International Hereditary Cancer Center, Pomerian Medical University, Szczecin, Poland

**Introduction**

Four founder mutations of the CHEK2 gene were detected in Polish population. Three of these mutations shorten protein del5395, IVS2 + 1G> A, 1100delC, and one is a single nucleotide substitution leading to amino acid substitution (missense mutation I157T). Mutations shortening protein are associated with a higher risk of breast cancer (approximately 3 times higher) than mutation I157T (risk increased about 1.5 times). Survival rating of patients with breast cancer and with one of the four mutations in the CHEK2 gene showed no significant differences compared to the group without the mutation. Reports in the literature evaluating the clinical trial of breast cancer in carriers of mutations in the CHEK2 gene are few. Shmidt proved the increased risk of death due to breast cancer in mutation carriers 1100delC and De Bock and Meyer found a higher risk of distant metastases. There is a lack of studies evaluating the incidence and type of recurrence in carriers of mutations in the CHEK2 gene in Polish population.

**Materials and methods**

The aim of the study was to evaluate the characteristics of clinico-pathological and clinical trial of breast cancers diagnosed in women with mutations in the CHEK2 gene. The study included 2,362 women with invasive breast cancer in stage I to IV diagnosed between 2001 to 2010 in Opolskie Centrum Onkologii, who marked four founder mutations in the CHEK2 gene (del5395, IVS2 + 1G> A, 1100delC, I157T). Patients diagnosed with ductal carcinoma and diagnosed with a mutation in BRCA1 or BRCA2 were excluded from the group. A rate and reproducible clinical features of diagnosed breast cancers were evaluated within a group. In the group of patients with stage I to IIIA there was evaluated an incidence of subsequent primary tumors, the frequency and location of recurrence. The patients were observed prospectively from the date of diagnosis of breast cancer to August 2016.

**Results**

The frequency of mutations in the CHEK2 gene within a group was 8.1%. Changes shortening protein was detected in 1.8% of women and missense change at 6.3%. In the group of patients up to the age of 50 mutations reducing the protein was found in 2.4% of patients compared to 1.6% over the age of 50. I157T mutation was more common over 50 (6.6% vs 5.2%). Clinicopathological features of diagnosed breast cancers have shown: the higher incidence of clinically advanced breast cancer in stage III and IV in women with a mutation I157T (34.7% vs 24.9%; p = 0.01), a higher incidence of positive progesterone receptor in the group of patients with mutations in the CHEK2 gene shortening protein (85.4% vs 67.4%; p = 0.02). In the group of patients in stage I to IIIA after radical local treatment recurrence of the disease was found in 20.6% of patients with mutations shortening protein, 14.2% of patients with a missense mutation and 17.1% of patients without the mutation. There were no significant differences in the location of recurrence besides the higher frequency of local recurrence in the case of mutations shortening protein (42.9% vs 12.3%; p = 0.02). There is no difference in the number of consecutive primary tumors (8.8%, 13.3% and 14.2%).

